# MIST (Modified Intubating Sequence for Transmissibility) Bundle for Infectious Diseases with Aerosol Hazard

**DOI:** 10.5811/westjem.2020.7.47473

**Published:** 2020-08-17

**Authors:** Jayaraj M. Balakrishnan, Sanjan Asanaru Kunju, William Wilson, Sachin Nayak Sujir, Rachana Bhat, K. E. Vandana

**Affiliations:** * Department of Emergency Medicine, Kasturba Medical College, Manipal, Manipal Academy of Higher Education, Manipal, Karnataka, India; † Department of Microbiology, Kasturba Medical College, Manipal, Manipal Academy of Higher Education, Manipal, Karnataka, India

## Abstract

The current global severe acute respiratory syndrome coronavirus 2 (SARS-CoV-2) pandemic has magnified the risk to healthcare providers when inititiating airway management, and safe tracheal intubation has become of paramount importance. Mitigation of risk to frontline providers requires airway management to be an orchestrated exercise based on training and purposeful simulation. Role allocation and closed-loop communication form the foundation of this exercise. We describe a methodical, 10-step approach from decision-making and meticulous drug and equipment choices to donning of personal protective equipment, and procedural concerns. This bundled approach will help reduce unplanned actions, which in turn may reduce the risk of aerosol transmission during airway management in resource-limited settings.

## BACKGROUND

The emergence of the severe acute respiratory syndrome coronavirus 2 (SARS-CoV-2), or COVID-19, pandemic has brought infections that are transmitted via droplet and aerosol under the spotlight.^1^ Infections such as influenza A subtype H1N1, Nipah virus infection, Ebola virus disease, and multidrug-resistant tuberculosis are equally contagious and pose a significant risk to healthcare professionals, especially those involved in airway management.^2^,^3^ Herin, we describe a step-by-step approach to endotracheal intubation of critically ill patients with suspected or confirmed COVID-19 and other airborne diseases with the goal of limiting the risk of exposure to healthcare providers.

## CONCEPTS

1. Rapid sequence intubation (RSI) and invasive mechanical ventilation are preferred. Non-invasive positive pressure ventilation (NIPPV) increases the risk of aerosol generation; NIPPV has been associated wth increased risk of healthcare worker infection and hence should be avoided.^4^–^6^
2. The care area is divided as follows:
  - Hot zone: A three-meter [9.85 feet] radius around the patient
  - Warm zone: The area between hot and cold zone where decontamination takes place
  - Cold zone: The outermost noncontaminated area.
3. The intubation team members are described in Table 1.
4. Encourage closed-loop communication.

**Population Health Research Capsule**

What do we already know about this issue?

Several protocols for intubation during the COVID-19 pandemic aim at reducing transmissibility of infections by using sophisticated equipment.

What was the research question?

How can we reduce the risk of aerosol hazards from infectious diseases transmitted during intubation?

What was the major finding of the study?

Execution of intubation in suspected aerosol-transmitted infections can be performed systematically in low-resource settings.

How does this improve population health?

The protocol is aimed at safeguarding healthcare professionals against aerosol hazards while performing airway interventions.

## STEPS OF MIST (Modified Intubating Sequence for Transmissibility) BUNDLE

**Figure 1:**
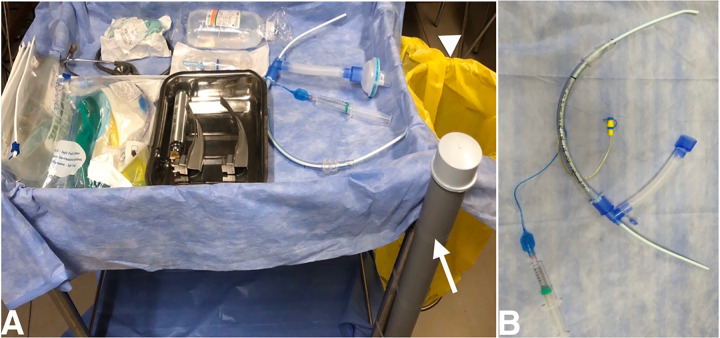
A. List of equipment required in intubation trolley. PVC pipe sealed at one end (white arrow) filled with 1% sodium hypochlorite solution is used for discarding the soiled bougie and yellow bag (arrow head) for discarding the soiled wastes. B. Intubation unit.

1. **Pre-assessment phase and pre-briefing phase – Cold Zone**
  Step a. Review patient clinical data to determine appropriateness of endotracheal intubation and mechanical ventilation for the patient.
  Step b. Team leader (TL) debriefs the intubation plan to the team to avoid unplanned and unarticulated actions.
  Step c. Infection control nurse (ICN) alerts team to any breach of protocol or infection control practice.
2. **Preparatory phase – Cold Zone**
  Step a. Use continous positive airway pressure mode with non-invasive ventilation (NIV) mask for preoxygenation. The registered respiratory therapist (RRT) assembles the ventilator with its circuit including preparation of the NIV mask with a viral filter and checks for possible leaks and disconnections. Ventilator settings: pressure support 0 centimeters of water, positive end expiratory pressure as per the requirement, and fraction of inspired oxygen to 100%. Deselect the apnea setting.
  Step b. Review the equipment required for intubation (Figure 1A) (Table 2); the registered nurse (RN) loads pre-calculated doses of RSI medications (Table 3).
  Step c. The assembly (Figure 1B) of the endotracheal tube (ETT) should be preset with a catheter mount containing a viral filter, and an inflation syringe with an intubating bougie.
3. **Preoxygenation Phase: Hot Zone**
  Step a: The RRT ensures wall-mounted suction unit is properly connected. A Yankauer suction connected to the wall-mounted suction unit should be available, but its usage should be judicious. The suction tip, if used, should be disposed of in a Ziploc bag.
  Step b: TL at the head of the bed places the NIV mask with the viral filter onto the patient and ensures proper sealing to avoid leak. The RRT “starts” the ventilator and preoxygenates until adequate oxygen saturation is attained. Avoid bag-valve mask for preoxygenation. Meticulous preoxygenation should be done for 3–5 minutes.
  Step c: RN ensures patent intravenous access, assesses the vitals periodically, and communicates them to the TL.
4. **Peri Induction phase: Hot Zone**
  Step a: RN administers the pre-calculated dose of the induction agent followed by the paralytic agent to the patient.
  Step b: Approrpiate patient positioning should be performed to maximise safe apnoea time.
5. **Peri-intubation Phase: Hot Zone**
  Step a: The RRT sets the ventilator on standby mode b after adequate paralysis and oxygen saturation is achieved.
  Step b: TL subsequently removes the NIV mask, which is disconnected from the ventilator by the RRT and placed in a Ziploc bag.
  Step c: TL performs laryngoscopy. During this time, the RRT is required to change the settings of the ventilator to “assist control mode ventilation.”. Once the vocal cords are visualized, the RRT hands over the intubating unit to the TL who should then pass the bougie between the cords under direct visualization. Video laryngoscope is a preferred choice for intubation of such patients, if available.
  Step d: The RRT assists in guiding the ET over the bougie and should subsequently inflate the cuff with the pre-filled air syringe.
  Step e: The RRT then removes the bougie and disposes of it in the preset disposition system (Figure 1A).
  Step f: The RRT then proceeds to connect the ET to the ventilator and convert the ventilator from standby to its preset settings. Simultaneously, the TL removes the laryngoscope and places it in a Ziploc bag.
  Step g: The RN confirms the position of the tube with five-point auscultation, following which the stethoscope should be disposed of in the Ziploc bag. End-tidal carbon dioxide confirmation is advised, if available.
6. **Post-intubation Phase: Hot Zone**
  Step a: Continue ventilation and monitor hemodynamics. Initiate early sedation and analgesia.
  Step b: Ensure all soiled equipment has been disposed of appropriately into the yellow bag for decontamination (Figure 1A).^8^
  Step c: The order of doffing and decontamination is TL, followed by the RN, and then the RRT who should be separately overviewed and monitored by the ICN.

## CONCLUSION

This sequence should guide healthcare professionals to minimize aerosol and droplet transmission during intubation and expedite better patient care. This approach does not involve significant resource intensification and can be done in resource-limited settings.

## Supplementary Information



## Figures and Tables

**Table 1:** Role Allocation and Personnel Details of intubation team.

S. No	Personnel	Stationed in	Responsibility
1	Team leader	Hot Zone (3-meter radius)	Performs tracheal intubation
2	Registered respiratory therapist	Hot Zone	Oversees airway and ventilator equipment
3	Registered nurse	Hot Zone	Ensures IV access and administers IV medications
4	Infection control nurse	Warm Zone	Oversees procedure and protocols

*IV*, intravenous; *TL*, Team Leader; *RRT*, Registered Respiratory Therapist; *RN*, Registered Nurse; *ICN*, Infection Control Nurse.

**Table 2:** List of equipment required in intubation trolley.

Equipment	Numbers
Intubation unit (Figure 1B)	1
Macintosh laryngoscope with size 3 and 4 blades in a sterile tray	1 each
iGel	2
Stethoscope	1
Cuffed ETT, size 7	1
ETT fixator	1
IV fluid with infusion set	1
Ziploc bags	5

*ETT*, endotracheal tube; *IV*, intravenous.

**Table 3:** List of drugs used in Intubation.

Drugs	Dose
Inducing agent	
Inj. etomidate or	0.3mg/kg IV
Inj. ketamine	1–2mg/kg IV
Paralytic agent	
Inj. rocuronium	1–1.2mg/kg IV

*Inj*, injection; *mg*, milligram; *kg*, kilogram; *IV*, intravenous.
