# Supplementary material for: MIST (Modified Intubating Sequence for Transmissibility) Bundle for Infectious Diseases with Aerosol Hazard
Source: West J Emerg Med. 2020 Aug 17;21(5):1076–9. doi: 10.5811/westjem.2020.7.47473 (PMC7514411; doi:10.5811/westjem.2020.7.47473)
Supplement: Supplementary file 1 [file wjem-21-1076-s001.pdf]

MIST BUNDLE CHECKLIST

COLD ZONE

- ❏ Determine the risk-benefit ratio of intubation
- ❏ Allocation of roles to the team members
- ❏ Use of closed-loop communication

Role of Registered Respiratory Therapist (RRT)

- ❏ Pre-set the ventilator for pre-oxygenation
- ❏ Ensure sturdy connection of ventilator tubing
- ❏ Equipment in intubation trolley
  - Intubation unit
  - Macintosh Laryngoscope with blade size 3 and 4 in a sterile tray
  - Video laryngoscope preferred (if available)
  - Stethoscope
  - Cuffed ET tube
  - ET Tube fixator
  - IV fluid with infusion set
  - Zip lock bags

Role of Registered Nurse (RN)

- ❏ Preload drugs with labelled syringes
- ❏ Drugs
  - Inj. Etomidate (0.3mg/kg body wt) or Inj. Ketamine (1-2 mg/kg body wt)
  - Inj. Rocuronium (1-1.2mg/kg body wt)
  - Vasopressor (if required)
  - Infusions for sedation and paralysis

PPE

- ❏ Donning according to standard principles

HOT ZONE

Role of Team Leader (TL)

- ❏ Proper mask seal with NIV + Optimize patient position
- ❏ Adequate pre-oxygenation for 3mins until saturation is >88%
- ❏ Remove NIV mask only after the ventilator is on standby mode
- ❏ Ensure visual passage of bougie through the vocal cords
- ❏ Secure tube with tube fixator
- ❏ Place the laryngoscope with tray into a zip lock bag

Role of Registered Respiratory Therapist (RRT)

- ❏ Ensure functioning of wall mounted suction
- ❏ Start ventilator for pre-oxygenation AFTER adequate mask seal
- ❏ Keep ventilator on standby mode after the pre-oxygenation
- ❏ Place the NIV mask in a zip lock bag
- ❏ Preset the ventilator to ACMV for ventilation after intubation
- ❏ Assist the TL with the intubating unit while passing the bougie through the vocal cord
- ❏ Inflate the cuff immediately after tracheal intubation
- ❏ Remove the bougie and discard into pre-set disposition system
- ❏ Confirm tube position by ETCO2 monitor or five point auscultation
- ❏ Start mechanical ventilation only after cuff inflation
- ❏ Ensure there is no leak

Role of Registered Nurse (RN)

- ❏ Ensure the patency of IV access
- ❏ Communicate the vitals periodically to the TL
- ❏ Administer the inducting and paralytic agent
- ❏ Initiate sedation and paralytic infusions

WARM ZONE

- ❏ Careful equipment disposal
- ❏ Decontamination of reusable equipment
- ❏ Remove PPE
  - Standard doffing procedure
  - Meticulous disposal
